# Nine out of ten samples were mistakenly switched by The Orang-utan Genome Consortium

**DOI:** 10.1038/s41597-022-01602-0

**Published:** 2022-08-12

**Authors:** Graham L. Banes, Emily D. Fountain, Alyssa Karklus, Robert S. Fulton, Lucinda Antonacci-Fulton, Joanne O. Nelson

**Affiliations:** 1 Wisconsin National Primate Research Center, University of Wisconsin–Madison, 1220 Capitol Court, Madison, WI 53715 USA; 2 School of Veterinary Medicine, University of Wisconsin–Madison, 2015 Linden Drive, Madison, WI 53706 USA; 3 The Orang-utan Conservation Genetics Project, Madison, WI 53715 USA; 4 McDonnell Genome Institute at Washington University, Washington University School of Medicine, 4444 Forest Park Avenue, Saint Louis, MO 63108 USA

**Keywords:** Genetics, Zoology, Evolution

## Abstract

The Sumatran orang-utan *(Pongo abelii)* reference genome was first published in 2011, in conjunction with ten re-sequenced genomes from unrelated wild-caught individuals. Together, these published data have been utilized in almost all great ape genomic studies, plus in much broader comparative genomic research. Here, we report that the original sequencing Consortium inadvertently switched nine of the ten samples and/or resulting re-sequenced genomes, erroneously attributing eight of these to the wrong source individuals. Among them is a genome from the recently identified Tapanuli *(P. tapanuliensis)* species: thus, this genome was sequenced and published a full six years prior to the species’ description. Sex was wrongly assigned to five known individuals; the numbers in one sample identifier were swapped; and the identifier for another sample most closely resembles that of a sample from another individual entirely. These errors have been reproduced in countless subsequent manuscripts, with noted implications for studies reliant on data from known individuals.

## Introduction

Alongside their publication of a Sumatran orang-utan *(Pongo abelii)* draft genome assembly in 2011, The Orang-utan Genome Consortium re-sequenced the genomes of ten additional unrelated wild-caught individuals – ostensibly five Sumatran and five Bornean *(P. pygmaeus)* orang-utans – using short-read Illumina sequencing^1^. Their manuscript, and its accompanying 297 Gb of sequence data, has since been cited more than 500 times. During the course of our own studies, however, we noted several inconsistencies between the data made available in the NCBI Sequence Read Archive and their accompanying metadata and descriptors in the paper.

We found no record of a sample with the identifier “KB5543”, for example, in the Frozen Zoo repository, the reported source of a sample attributed to the orang-utan, Louis. The closest match in their database to this ID was for another sample, “15543”, which derived from a different individual. We also observed that the identifier “KB9528”, as reported for the orang-utan Baldy in the manuscript’s Tables S4-1, was catalogued as a sample from an “African pig” – though, in a supplemental file, it was correctly denoted as KB9258, which derived from another orang-utan. The sample identifier “SB550”, as reported for the orang-utan Doris, appeared to reference a studbook number (*i.e*. “SB”) that belonged to another sequenced orang-utan, Sibu. The sex reported for five individuals also contradicted their known sexes, as had been recorded in contemporary studbook records^2^, plus differed from the sexes assigned to each sample in Locke *et al*.’s supplementary data.

Thus, we were driven to reconsider the identities of each genome’s source individual, through re-analysis of the published data combined with new molecular studies. Herein, we report that nine of the ten samples and/or published genomes were erroneously labelled in the original *Nature* publication. We present the corrected data and discuss the implications for other published works.

## Methods

We first mapped the re-sequencing reads of all 10 Locke *et al*. whole genomes, plus those previously published from 27 conspecifics^3,4^, to the latest iteration of the (female) orang-utan reference genome (ponAbe3^5^). To this, we had concatenated a recent orang-utan Y chromosome assembly^6^. Using the idxtools function in samtools 1.14^7^, we inferred sex by comparing the ratios to which sequence reads were mapped against the X and Y chromosomes. Following two rounds of bootstrapped base recalibration, we then jointly called genotypes with GATK 4.1.8.0^8^, all as previously described^9^. We randomly sampled 1,000,000 biallelic autosomal SNPs with no missing genotypes and ≥5% minor allele frequency (MAF), pruned linked loci in PLINK^10^ (–indep-pairwise 50 10 0.1), and assigned populations in ADMIXTURE 1.39^11^ as supervised with provenance data reported for the conspecifics^3,4^ (K = 3).

**Fig. 1:**
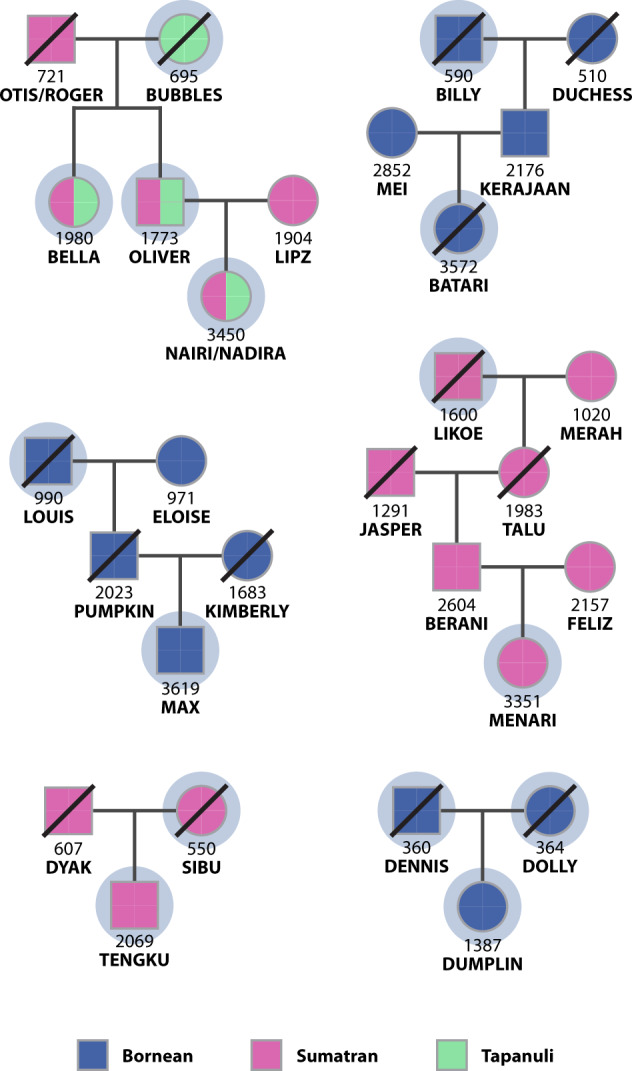
Genogram depicting relationships between orang-utans sampled by Locke *et al*. and known relatives assayed in this study. Orang-utans used in the kinship analysis (Table 1) are circled. The species affiliations of uncircled orang-utans reflect those purported by contemporary studbook records.

Additionally, we sampled and assayed eight orang-utans known to be first, second or third-degree relatives of seven of those purportedly sequenced by Locke *et al*., using the Illumina iScan Multi-Ethnic Global Array, also as previously described^12^. The reproduction of those seven, and thus these known relationships, had been contemporaneously recorded^2^ (Fig. 1). To convert the microarray intensity data to variant calls, we mapped the probe flank sequences to ponAbe3 (using --fasta-flank) and exported genotypes (--sam-flank) with the bcftoools^7^ plugin gtc2vcf (https://github.com/freeseek/gtc2vcf), subject to the following filter parameters: meanR_AB < 0.2, meanR_AA < 0.2, meanR_BB < 0.2, Cluster_Sep < 0.35, meanTHETA_AA > 0.3, meanTHETA_BB < 0.7, meanTHETA_AB < 0.3 and > 0.7, devTHETA_AA > 0.025, devTHETA_AB ≥ 0.07, devTHETA_BB > 0.025 and GenTrain_Score < 0.7. We then re-genotyped all 37 whole genomes at each of the resulting loci, as previously described^9^; merged these with the microarray genotype VCF, and LD-pruned and MAF-filtered biallelic SNPs precisely as aforementioned. With a view to avoiding the spurious kinship associations that typify highly structured data, we then bootstrapped ADMIXTURE’s cross-validation procedure to infer the most suitable *K* (trialling 1 through 10) before estimating kinship coefficients (Φ_ij_) in REAP^13^.

We adopted a tri-fold method to confirm each sample’s identity. Identities were first inferred with an exclusionary approach, from computed (versus known and reported) sex and species. Each was then confirmed, where available, when observed kinship coefficients resembled those expected from known relationships. Third, we reviewed the historical biomaterial records retained by the Frozen Zoo, the original source of the samples, plus notes from the Laboratory Information System (LIMS) retained at Washington University in Saint Louis, where the samples were originally sequenced. Identity was assigned to a given sample when all these factors concorded.

## Results

**Fig. 2:**
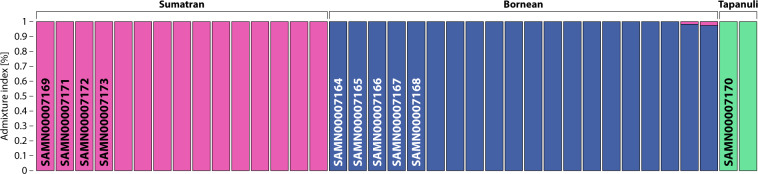
Ancestry proportions of the Locke *et al*. orang-utans, as supervised with provenance data from 27 conspecifics sampled across the natural range of the genus^3,4^. Sample SAMN000007170 derives from a Tapanuli orang-utan.

We observed X:Y sequence ratios in known males to range from 0.369–0.569 (mean 0.476) and in females from 4.114 to 5.827 (mean 4.973). From this, we interpreted that sex had been incorrectly assigned by Locke *et al*. to the sample SAMN00007170. This sample was inferred to be female (4.170) and thus cannot have derived from Baldy as purported. The species of each sample was correctly reported, though we inferred that the sample SAMN00007170 derived from a Tapanuli orang-utan (Fig. 2). This species was not formally described until 2017^4^.

Kinship analyses linked one or more known relatives to seven of the ten samples sequenced by Locke *et al*. Specifically, we linked Billy to his granddaughter, Batari (observed/expected kinship coefficients: 0.116/0.125); Dumplin to her parents, Dennis (0.162/0.25) and Dolly (0.19/0.25); Sibu to her son, Tengku (0.246/0.25); Louis to his grandson, Max (0.082/0.125); Likoe to his great granddaughter, Menari (0.063/0.063); and Bubbles to her son, Oliver (0.233/0.25), daughter, Bella (0.242/0.25) and granddaughter, Nairi/Nadira (0.036/0.125). Admixture (K = 3) and kinship were inferred from a total of 1,132,210 biallelic SNPs.

We assigned identity to the remaining three samples as molecular sex, sample and LIMS records were all concordant, and as the relatedness data had excluded other possible candidates. Table 1 corrects the record as originally presented in *Nature*.

**Table 1:** Corrected identities and metadata for the samples sequenced and published by Locke *et al*.^1^, as deposited in the NCBI BioSample database.

BioSample ID	Reported identities and metadata					Corrected/validated identities and metadata						Known relative and inferred kinship					
	Lab ID	ISB	Name	Sp.	Sex	Lab ID	ISB	Name	Sp.	Sex	X:Y	ISB	δ_0_	δ_1_	δ_2_	Exp. Φ_ij_	Φ_ij_
SAMN00007164	KB5404	590	Billy	B	F	KB5404	356	Dinah	B	F	4.556	—	—	—	—	—	—
SAMN00007165	KB4204	364	Dolly	B	M	KB4204	590	Billy	B	M	0.506	3572	0.655	0.226	0.119	0.125	0.116
SAMN00007166	KB5406	356	Dinah	B	F	KB5406	364	Dolly	B	F	5.015	1387	0.432	0.376	0.192	0.250	0.190
SAMN00007167	KB5405	360	Dennis	B	M	KB5405	360	Dennis	B	M	0.547	1387	0.411	0.530	0.060	0.250	0.162
SAMN00007168	KB5543	990	Louis	B	M	—	990	Louis	B	M	0.464	3619	0.823	0.025	0.152	0.125	0.082
SAMN00007169	KB5883	550	Sibu	S	M	KB5883	1600	Likoe	S	M	0.444	3351	0.971	0.000	0.221	0.063	0.063
SAMN00007171	KB4661	695	Bubbles	S	M	KB4661	732	Baldy	S	M	0.432	—	—	—	—	—	—
SAMN00007172	KB4361	1600	Likoe	S	F	KB4361	53	Doris	S	F	4.625	—	—	—	—	—	—
SAMN00007173	SB550	53	Doris	S	F	—	550	Sibu	S	F	4.264	2069	0.313	0.390	0.298	0.250	0.246
SAMN00007170	KB9528	732	Baldy	S	M	KB9258	695	Bubbles	T	F	4.170	1980	0.200	0.632	0.168	0.250	0.242
												1773	0.186	0.696	0.118	0.250	0.233
												3450	0.771	0.229	0.000	0.125	0.036

Originally reported data are reproduced from Locke *et al*.’s Tables S4-1. “ISB” denotes International Studbook Number; for species, “B” indicates Bornean *(P. pygmaeus)*, “S” indicates Sumatran *(P. abelii)*, and “T” indicates Tapanuli *(P. tapanuliensis)*. The X:Y ratios noted for sex are those inferred, as detailed, from each mapped BAM file. “Lab ID” is the internal identifier used by the sequencing facility, as variously recorded by Locke *et al*. Relatedness is reported as the probability that each sequenced orang-utan shares 0, 1 and 2 alleles identical by descent with a known relative (*i.e*. δ_0_, δ_1_, and δ_2_, respectively), plus the expected/theoretical (Exp.) and computed kinship coefficient (Φ_ij_).

## Discussion

Because Locke *et al*. focused solely on genome content, their discrepancies have no bearing on the accuracy of their data or their manuscript’s published findings. These errors have had considerable impact on other studies that utilized the published data, however, particularly those dependent on using data from known individuals. Three of our co-authors (GLB, EDF, AK) write from first-hand experience: reliant on tables and metadata from the original *Nature* publication, we came perilously close to incorrectly reporting that Baldy, a male orang-utan who lived at the Sacramento Zoo, was the first of the recently described Tapanuli species to be captured and exported from a wild population – a full five decades before his species’ formal description. On the contrary, this dubious honour belongs to Bubbles, a female orang-utan who lived at the San Diego Zoo. Though beyond the scope of our manuscript, the implications of this switch have not escaped our attention: principally, that Bubbles produced eight Sumatran *x* Tapanuli hybrid descendants, who were previously thought to be Sumatran. The genetic integrity of the captive population is therefore unexpectedly compromised, as we present in detail in a manuscript that is currently under review.

Though we eventually caught these errors, others did not. Mattle-Greminger *et al*. (2018) reproduced eight erroneous sample identities in their paper, meaning each of the genomes they analysed were from different animals than reported^14^. Sudmant *et al*. (2013) reproduced seven such errors, thus also misattributing samples^15^. Neither Ma *et al*. (2013) nor Beeravolu *et al*. (2018) recognized that the sample identities were wrong, though as they reported only sample IDs (versus animal identities), no corrections to their manuscripts are warranted^16,17^. As sample identities are normally only reported in supplemental data – which is not always indexed by search engines – we cannot easily ascertain the full extent to which papers citing Locke *et al*. have reproduced these errors.

Given these findings and implications, we have corrected the samples’ identities in the NCBI BioSample database. Tables detailing the revisions made are included in Supplementary File 1. We respectfully ask that those utilizing these updated identities cite this article in *Scientific Data*, in addition to the Correction concurrently published in *Nature*.

## Supplementary information

Supplementary File 1

## Data Availability

Locke *et al*. deposited their sequence reads in the Short Read Archive under the accession codes detailed in their Supplementary Information^1^. Our microarray data from known relatives have been deposited in Figshare in their original IDAT format^18^.

## Code availability

No custom code was used to generate or process the data described in the manuscript.
