# Supplementary material for: Nine out of ten samples were mistakenly switched by The Orang-utan Genome Consortium
Source: Sci Data. 2022 Aug 12;9:485. doi: 10.1038/s41597-022-01602-0 (PMC9374732; doi:10.1038/s41597-022-01602-0)
Supplement: Supplementary file 1 [file 41597_2022_1602_MOESM1_ESM.docx]

**Supplementary Table 1.** Revised metadata for the NCBI BioSample accession code SAMN00007164.

| **SAMN00007164** | | | | |
| --- | --- | --- | --- | --- |
|  | **ORIGINAL METADATA** | | **REVISED METADATA** | |
| **Name** | Pongo pygmaeus KB5404, Bornean female | | Pongo pygmaeus, Bornean female, ISB 356 Dinah | |
| **Organism** | Pongo pygmaeus (Bornean orangutan) | | No change | |
| **Description** | Gender : F Sample Type : genomic dna | | KB5404, Bornean female | |
| **BIOSAMPLE IDENTIFIERS** | | | | |
| **BioSample:** | SAMN00007164 | **BioSample:** | | No change |
| **Sample name:** | 45417 | **Sample name:** | | No change |
| **SRA:** | SRS009463 | **SRA:** | | No change |
| **BIOSAMPLE ATTRIBUTES** | | | | |
| ***No attributes*** |  | **sex** | | Female |
|  |  | **individual_id** | | DINAH/DINA |
|  |  | **source_material_id** | | KB5404 |
|  |  | **sample_name** | | 45417 |
|  |  | **sample_type** | | Genomic DNA |
|  |  | **sequenced_by** | | WUGSC |
|  |  | **stud_book_number** | | ISB_356 |
|  |  | **birth_date** | | 1955-01-01 ± 2Y |
|  |  | **birth_location** | | WILD |
|  |  | **death_date** | | 1986-10-06 |
|  |  | **karyotype** | | K |
|  |  | **ref_biomaterial** | | This manuscript |

**Supplementary Table 2.** Revised metadata for the NCBI BioSample accession code SAMN00007165.

| **SAMN00007165** | | | | |
| --- | --- | --- | --- | --- |
|  | **ORIGINAL METADATA** | | **REVISED METADATA** | |
| **Name** | Pongo pygmaeus KB4204, Bornean male | | Pongo pygmaeus, Bornean male, ISB 590 Billy | |
| **Organism** | Pongo pygmaeus (Bornean orangutan) | | No change | |
| **Description** | KB4204, Bornean male PPAR-070502_KB4204 | | KB4204, Bornean male | |
| **BIOSAMPLE IDENTIFIERS** | | | | |
| **BioSample:** | SAMN00007165 | **BioSample:** | | No change |
| **Sample name:** | 45418 | **Sample name:** | | No change |
| **SRA:** | SRS009464 | **SRA:** | | No change |
| **BIOSAMPLE ATTRIBUTES** | | | | |
| **sex** | male | **sex** | | No change |
| **individual_id** | KB4204 | **individual_id** | | BILLY |
|  |  | **source_material_id** | | KB4204 |
|  |  | **sample_name** | | 45418 |
|  |  | **misc_param** | | PPAR-070502_KB4204 |
|  |  | **sequenced_by** | | WUGSC |
|  |  | **stud_book_number** | | ISB_590 |
|  |  | **birth_date** | | 1962-01-02 ± 2Y |
|  |  | **birth_location** | | WILD |
|  |  | **death_date** | | 2000-06-22 |
|  |  | **karyotype** | | K |
|  |  | **ref_biomaterial** | | This manuscript |

**Supplementary Table 3.** Revised metadata for the NCBI BioSample accession code SAMN00007166.

| **SAMN00007166** | | | | |
| --- | --- | --- | --- | --- |
|  | **ORIGINAL METADATA** | | **REVISED METADATA** | |
| **Name** | Pongo pygmaeus KB5406, Bornean female | | Pongo pygmaeus, Bornean female, ISB 364 Dolly | |
| **Organism** | Pongo pygmaeus (Bornean orangutan) | | No change | |
| **Description** | KB5406, Bornean female GPPAU-070502_KB5406 | | KB5406, Bornean female | |
| **BIOSAMPLE IDENTIFIERS** | | | | |
| **BioSample:** | SAMN00007166 | **BioSample:** | | No change |
| **Sample name:** | 45456 | **Sample name:** | | No change |
| **SRA:** | SRS009465 | **SRA:** | | No change |
| **BIOSAMPLE ATTRIBUTES** | | | | |
| **sex** | female | **sex** | | No change |
| **Individual_id** | KB5406 | **individual_id** | | DOLLY |
|  |  | **source_material_id** | | KB5406 |
|  |  | **sample_name** | | 45456 |
|  |  | **misc_param** | | GPPAU-070502_KB5406 |
|  |  | **sequenced_by** | | WUGSC |
|  |  | **stud_book_number** | | ISB_364 |
|  |  | **birth_date** | | 1957-01-01 ± 2Y |
|  |  | **birth_location** | | WILD |
|  |  | **death_date** | | 1993-05-18 |
|  |  | **karyotype** | | K |
|  |  | **ref_biomaterial** | | This manuscript |

**Supplementary Table 4.** Revised metadata for the NCBI BioSample accession code SAMN00007167.

| **SAMN00007167** | | | | |
| --- | --- | --- | --- | --- |
|  | **ORIGINAL METADATA** | | **REVISED METADATA** | |
| **Name** | Pongo pygmaeus KB5405, Bornean male | | Pongo pygmaeus, Bornean male, ISB 360 Dennis | |
| **Organism** | Pongo pygmaeus (Bornean orangutan) | | No change | |
| **Description** | KB5405, Bornean male PPAT-070502_KB5405_Tube1 | | KB5405, Bornean male | |
| **BIOSAMPLE IDENTIFIERS** | | | | |
| **BioSample:** | SAMN00007167 | **BioSample:** | | No change |
| **Sample name:** | 45457 | **Sample name:** | | No change |
| **SRA:** | SRS009466 | **SRA:** | | No change |
| **BIOSAMPLE ATTRIBUTES** | | | | |
| **sex** | male | **sex** | | No change |
| **Individual_id** | KB5405 | **individual_id** | | DENNIS |
|  |  | **source_material_id** | | KB5405 |
|  |  | **sample_name** | | 45457 |
|  |  | **misc_param** | | PPAT-070502_KB5405_Tube1 |
|  |  | **sequenced_by** | | WUGSC |
|  |  | **stud_book_number** | | ISB_360 |
|  |  | **birth_date** | | 1955-01-01 ± 2Y |
|  |  | **birth_location** | | WILD |
|  |  | **death_date** | | 1988-12-05 |
|  |  | **karyotype** | | K |
|  |  | **ref_biomaterial** | | This manuscript |

**Supplementary Table 5.** Revised metadata for the NCBI BioSample accession code SAMN00007168.

| **SAMN00007168** | | | | |
| --- | --- | --- | --- | --- |
|  | **ORIGINAL METADATA** | | **REVISED METADATA** | |
| **Name** | KB5543, Bornean male | | Pongo pygmaeus, Bornean male, ISB 990 Louis | |
| **Organism** | Pongo pygmaeus (Bornean orangutan) | | No change | |
| **Description** | KB5543, Bornean male PPAV-070502_KB5543 | | Bornean male | |
| **BIOSAMPLE IDENTIFIERS** | | | | |
| **BioSample:** | SAMN00007168 | **BioSample:** | | No change |
| **Sample name:** | 45419 | **Sample name:** | | No change |
| **SRA:** | SRS009467 | **SRA:** | | No change |
| **BIOSAMPLE ATTRIBUTES** | | | | |
| **sex** | male | **sex** | | No change |
| **Individual_id** | KB5543 | **individual_id** | | LOUIS |
|  |  | **source_material_id** | | Unknown |
|  |  | **sample_name** | | 45419 |
|  |  | **misc_param** | | PPAV-070502_KB5543 |
|  |  | **sequenced_by** | | WUGSC |
|  |  | **stud_book_number** | | ISB_990 |
|  |  | **birth_date** | | 1964-01-01 ± 2Y |
|  |  | **birth_location** | | WILD |
|  |  | **death_date** | | 1998-02-24 |
|  |  | **karyotype** | | K |
|  |  | **ref_biomaterial** | | This manuscript |

**Supplementary Table 6.** Revised metadata for the NCBI BioSample accession code SAMN00007169.

| **SAMN00007169** | | | | |
| --- | --- | --- | --- | --- |
|  | **ORIGINAL METADATA** | | **REVISED METADATA** | |
| **Name** | Pongo abelii KB5883, Sumatran male | | Pongo abelii, Sumatran male, ISB 1600 Likoe | |
| **Organism** | Pongo abelii (Sumatran orangutan) | | No change | |
| **Description** | KB5883, Sumatran male PPAO-070502_KB5883 | | KB5883, Sumatran male | |
| **BIOSAMPLE IDENTIFIERS** | | | | |
| **BioSample:** | SAMN00007169 | **BioSample:** | | No change |
| **Sample name:** | 45437 | **Sample name:** | | No change |
| **SRA:** | SRS009468 | **SRA:** | | No change |
| **BIOSAMPLE ATTRIBUTES** | | | | |
| **sex** | male | **sex** | | No change |
| **Individual_id** | KB5883 | **individual_id** | | LIKOE |
|  |  | **source_material_id** | | KB5883 |
|  |  | **sample_name** | | 45437 |
|  |  | **misc_param** | | PPAO-070502_KB5883 |
|  |  | **sequenced_by** | | WUGSC |
|  |  | **stud_book_number** | | ISB_1600 |
|  |  | **birth_date** | | 1964-01-01 ± 2Y |
|  |  | **birth_location** | | WILD |
|  |  | **death_date** | | 1987-01-14 |
|  |  | **karyotype** | | K |
|  |  | **ref_biomaterial** | | This manuscript |

**Supplementary Table 7.** Revised metadata for the NCBI BioSample accession code SAMN00007170.

| **SAMN00007170** | | | | |
| --- | --- | --- | --- | --- |
|  | **ORIGINAL METADATA** | | **REVISED METADATA** | |
| **Name** | KB9258, Sumatran male | | Pongo tapanuliensis, Tapanuli female, ISB 695 Bubbles | |
| **Organism** | Pongo abelii (Sumatran orangutan) | | Pongo tapanuliensis (Tapanuli orang-utan) | |
| **Description** | KB9258, Sumatran male PPAP-070502_KB9258 | | KB9258, Tapanuli female | |
| **BIOSAMPLE IDENTIFIERS** | | | | |
| **BioSample:** | SAMN00007170 | **BioSample:** | | No change |
| **Sample name:** | 45458 | **Sample name:** | | No change |
| **SRA:** | SRS009469 | **SRA:** | | No change |
| **BIOSAMPLE ATTRIBUTES** | | | | |
| **sex** | male | **sex** | | Female |
| **Individual_id** | KB9258 | **individual_id** | | BUBBLES |
|  |  | **source_material_id** | | KB9258 |
|  |  | **sample_name** | | 45458 |
|  |  | **misc_param** | | PPAP-070502_KB9258 |
|  |  | **sequenced_by** | | WUGSC |
|  |  | **stud_book_number** | | ISB_695 |
|  |  | **birth_date** | | 1963-01-01 ± 2Y |
|  |  | **birth_location** | | WILD |
|  |  | **death_date** | | 1995-04-10 |
|  |  | **karyotype** | | K |
|  |  | **ref_biomaterial** | | This manuscript |

**Supplementary Table 8.** Revised metadata for the NCBI BioSample accession code SAMN00007171.

| **SAMN00007171** | | | | |
| --- | --- | --- | --- | --- |
|  | **ORIGINAL METADATA** | | **REVISED METADATA** | |
| **Name** | Pongo abelii KB4661, Sumatran male | | Pongo abelii, Sumatran male, ISB 732 Baldy | |
| **Organism** | Pongo abelii (Sumatran orangutan) | | No change | |
| **Description** | Pongo abelii KB4661, Sumatran male PPAN-070502_KB4661 | | KB4661, Sumatran male | |
| **BIOSAMPLE IDENTIFIERS** | | | | |
| **BioSample:** | SAMN00007171 | **BioSample:** | | No change |
| **Sample name:** | 45459 | **Sample name:** | | No change |
| **SRA:** | SRS009470 | **SRA:** | | No change |
| **BIOSAMPLE ATTRIBUTES** | | | | |
| **sex** | male | **sex** | | No change |
| **Individual_id** | KB4661 | **individual_id** | | BALDY |
|  |  | **source_material_id** | | KB4661 |
|  |  | **sample_name** | | 45459 |
|  |  | **misc_param** | | PPAN-070502_KB4661 |
|  |  | **sequenced_by** | | WUGSC |
|  |  | **stud_book_number** | | ISB_732 |
|  |  | **birth_date** | | 1960-01-01 ± 2Y |
|  |  | **birth_location** | | WILD |
|  |  | **death_date** | | 1992-11-14 |
|  |  | **karyotype** | | K |
|  |  | **ref_biomaterial** | | This manuscript |

**Supplementary Table 9.** Revised metadata for the NCBI BioSample accession code SAMN00007172.

| **SAMN00007172** | | | | |
| --- | --- | --- | --- | --- |
|  | **ORIGINAL METADATA** | | **REVISED METADATA** | |
| **Name** | KB4361, Sumatran female | | Pongo abelii, Sumatran female, ISB 53 Doris | |
| **Organism** | Pongo abelii (Sumatran orangutan) | | Pongo abelii (Sumatran orangutan) | |
| **Description** | KB4361, Sumatran female PPAM-070502_KB4361 | | KB4361, Sumatran female | |
| **BIOSAMPLE IDENTIFIERS** | | | | |
| **BioSample:** | SAMN00007172 | **BioSample:** | | No change |
| **Sample name:** | 45420 | **Sample name:** | | No change |
| **SRA:** |  | **SRA:** | | No change |
| **BIOSAMPLE ATTRIBUTES** | | | | |
| **sex** | female | **sex** | | No change |
| **Individual_id** | KB4361 | **individual_id** | | DORIS |
|  |  | **source_material_id** | | KB4361 |
|  |  | **sample_name** | | 45420 |
|  |  | **misc_param** | | PPAM-070502_KB4361 |
|  |  | **sequenced_by** | | WUGSC |
|  |  | **stud_book_number** | | ISB_53 |
|  |  | **birth_date** | | 1943-01-01 ± 2Y |
|  |  | **birth_location** | | WILD |
|  |  | **death_date** | | 1981-08-27 |
|  |  | **karyotype** | | K |
|  |  | **ref_biomaterial** | | This manuscript |

**Supplementary Table 10.** Revised metadata for the NCBI BioSample accession code SAMN00007173.

| **SAMN00007173** | | | | |
| --- | --- | --- | --- | --- |
|  | **ORIGINAL METADATA** | | **REVISED METADATA** | |
| **Name** | Pongo abelii SB550, Sumatran female | | Pongo abelii, Sumatran female, ISB 550 Sibu | |
| **Organism** | Pongo abelii (Sumatran orangutan) | | No change | |
| **Description** | SB550, Sumatran female PPAQ-070502_SB550 | | Sumatran female | |
| **BIOSAMPLE IDENTIFIERS** | | | | |
| **BioSample:** | SAMN00007173 | **BioSample:** | | No change |
| **Sample name:** | 45438 | **Sample name:** | | No change |
| **SRA:** | SRS009472 | **SRA:** | | No change |
| **BIOSAMPLE ATTRIBUTES** | | | | |
| **sex** | female | **sex** | | No change |
| **Individual_id** | SB550 | **individual_id** | | SIBU |
|  |  | **source_material_id** | | Unknown |
|  |  | **sample_name** | | 45438 |
|  |  | **misc_param** | | PPAQ-070502_SB550 |
|  |  | **sequenced_by** | | WUGSC |
|  |  | **stud_book_number** | | ISB_550 |
|  |  | **birth_date** | | 1956-01-01 ± 2Y |
|  |  | **birth_location** | | WILD |
|  |  | **death_date** | | 2004-12-08 |
|  |  | **karyotype** | | K |
|  |  | **ref_biomaterial** | | This manuscript |
